# *Bacillus anthracis *genome organization in light of whole transcriptome sequencing

**DOI:** 10.1186/1471-2105-11-S3-S10

**Published:** 2010-04-29

**Authors:** Jeffrey Martin, Wenhan Zhu, Karla D Passalacqua, Nicholas Bergman, Mark Borodovsky

**Affiliations:** 1 DOE Joint Genome Institute, Walnut Creek, CA 94598, USA; 2 School of Biology, Georgia Tech, Atlanta, GA, 30332, USA; 3 National Biodefense Analysis and Countermeasures Center, Frederick, MD 21702, USA; 4 Wallace H. Coulter Department of Biomedical Engineering and School of Computational Science and Engineering, Georgia Tech, Atlanta, GA, 30332, USA; 5 Center for Bioinformatics and Computational Genomics, Georgia Tech, Atlanta, GA, 30332, USA

## Abstract

Emerging knowledge of whole prokaryotic transcriptomes could validate a number of theoretical concepts introduced in the early days of genomics. What are the rules connecting gene expression levels with sequence determinants such as quantitative scores of promoters and terminators? Are translation efficiency measures, e.g. codon adaptation index and RBS score related to gene expression? We used the whole transcriptome shotgun sequencing of a bacterial pathogen *Bacillus anthracis *to assess correlation of gene expression level with promoter, terminator and RBS scores, codon adaptation index, as well as with a new measure of gene translational efficiency, average translation speed. We compared computational predictions of operon topologies with the transcript borders inferred from RNA-Seq reads. Transcriptome mapping may also improve existing gene annotation. Upon assessment of accuracy of current annotation of protein-coding genes in the *B. anthracis *genome we have shown that the transcriptome data indicate existence of more than a hundred genes missing in the annotation though predicted by an *ab initio *gene finder. Interestingly, we observed that many pseudogenes possess not only a sequence with detectable coding potential but also promoters that maintain transcriptional activity.

## Background

Significant efforts in the last two decades were directed to developing methods for genomic sequence functional interpretation, particularly, the means for predicting exact locations of transcribed and translated regions and levels of RNA and protein expression. At this time we were armed with technology able to sequence a whole prokaryotic transcriptome of a bacterial pathogen *Bacillus anthracis *and map RNA-Seq reads to genome.

We have used RNA-Seq reads mapped to genomic sequence as an input of an HMM based algorithm for parsing *B. anthracis *genomic sequence into a sequence of transcribed regions. Thus determined regions provided evidence for RNA transcription for majority of annotated genes. In addition we have seen RNA transcription from loci harboring mutated and presumably dysfunctional genes, the pseudogenes, as well as from a few regions containing genes predicted by an *ab initio *gene finder, GeneMarkS [1], but not annotated yet. We have detected that expression levels of adjacent genes located within borders of predicted transcripts correlate well, while adjacent genes residing in different transcripts do not exhibit any correlation. We have analyzed whether the evidence derived from RNA-Seq mapped reads would support operon predictions made by OperonDB [2].

Other questions we have addressed here were to find out if a score of a promoter site identified upstream to predicted transcription start site (TSS) correlates with gene expression level of the downstream gene. Also, is there a correlation of the gene expression level with the scores of the hairpin and tail of the downstream transcription terminator? Do locations of transcription end sites (TES) predicted by TransTermHP [3] appear in genomic sequence close to locations of the TES sites predicted from the mapped RNA-Seq reads?

Codon adaptation index (CAI) introduced by Sharp and Li [4] as a predictor of gene expression level has been broadly used, evaluated and modified [5-13]. There is an increasing evidence that evolution of genome GC content is the major driving force of evolution of genome wide codon usage [14-17]. Therefore, in a given genome room for codon usage variation between genes with lower and higher expression is narrower in genomes with high as well as low GC content. However, the synonymous codon most frequently used in its group as defined for the genome wide gene set is not always the same as the synonymous codons most frequently used in its group in a subset of genes with high expression. Therefore, selection of reference groups of highly expressed genes remains a rational approach for developing sequence dependent predictors of gene expression. *B. anthracis*, a species with relatively low, 35.4%, genome GC content, has 95 tRNA genes, and undergoes a strong selection for codon usage bias as indicated by the S index value 2.045 [11]. We have introduced a new measure of efficiency of translation, the average translation speed of a gene, an ATS index. We have shown values of both CAI and ATS correlate with gene expression levels. Finally, an existence of correlation between the score of ribosomal binding site (RBS) and gene expression level was not demonstrated so far. We have revisited this issue focusing our attention on the genes that are supposed to be the first genes in operons.

## Materials and methods

### Preparation of RNA-Seq data

In order to investigate the transcriptional landscapes of *B. anthracis *under a variety of stress conditions, *B. anthracis *str. 'Ames Ancestor' was subjected to the following growth stresses: (i) cold shock; (ii) osmotic shock as imposed by 0.75 M sodium chloride (NaCl); and (iii) 6% ethanol shock. For all experiments, the 'Ames Ancestor' strain was grown from a fresh colony on a blood agar plate in rich medium (LB broth) at 37°C to mid-exponential phase (optical density 600 nm (OD600) of 0.4-0.5), subjected to the aforementioned stresses, and then RNA was harvested by phenol:chloroform extraction as reported previously [18]. A control sample with no treatment was collected for baseline transcriptional activity. All experiments were performed in four biological replicates. For cold stress, cells were grown as above, and an equal volume of fresh medium that had been chilled overnight at 4°C was added. The cells were incubated with shaking at 17°C for 10 minutes followed by immediate RNA extraction. For NaCl stress, cells were grown as above, and an equal volume of 1.5 M NaCl LB medium was added for a final NaCl concentration of 0.75 M. RNA was harvested after 10 minutes of growth at 37°C with shaking. For ethanol stress, bacteria were grown as above, and an equal volume of pre-warmed (37°C) medium with 12% ethanol was added to the growing cells for a final concentration of 6% ethanol. Cells were grown with shaking at 37°C for 10 minutes and then RNA was harvested. Stress parameters were chosen such that the imposed stress was not entirely lethal to the bacteria in pilot experiments, but which would be strong enough to impose a fast and robust transcriptional survival response. RNA samples were subjected to rRNA depletion and SOLiD sequencing as described in [18]. ]. Library creation for SOLiD sequencing was done using the Applied Biosystems Whole Transcriptome Library Preparation Protocol and reagents.  SOLiD sequencing was performed at EDGE Biosystems, Gaithersburg, MD (http://www.edgebio.com). SOLiD data were mapped to the *B. anthracis *Ames Ancestor genome using SOCS [19], with a maximum tolerance of 5 color mismatches between each 50-color sequence read and the reference genome. A full description of these experiments and their biological implications is in preparation (KDP and NB, in progress).

### HMM model and the Viterbi algorithm for inferring transcripts from RNA-Seq

**Figure 1:**
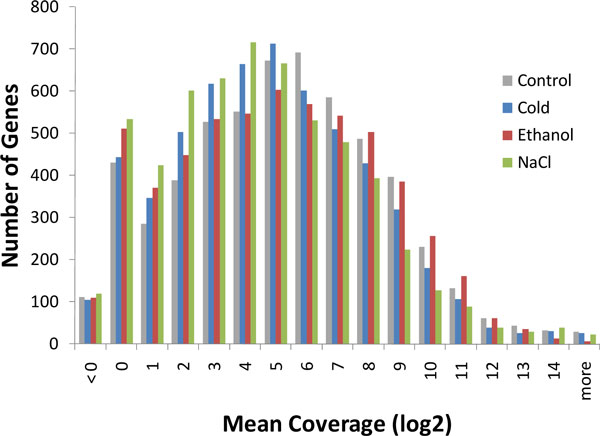
**Distributions of read coverage values (averaged within a gene) for four growth conditions**.

Determination of precise transcript boundaries and expression levels from mapped RNA-Seq reads is not a trivial task [18,20]. There are many confounding factors that make it difficult to pinpoint the borders between transcribed and non-transcribed regions. We use the term 'coverage' to designate a number of reads mapped to a particular genomic position. Since reads are generated at random significant deviations from uniformity in coverage are commonly observed. Also, transcripts are subject to degradation, which further contributes to noise in coverage data. Notably, a gene expression level is specific to the cell growth condition. Many genes exhibit low average coverage; the average gene coverage distribution (Figure 1) shows that 15-18% of the 5,661 genes have an average coverage ≤ 2 depending on the growth condition.

**Figure 2:**
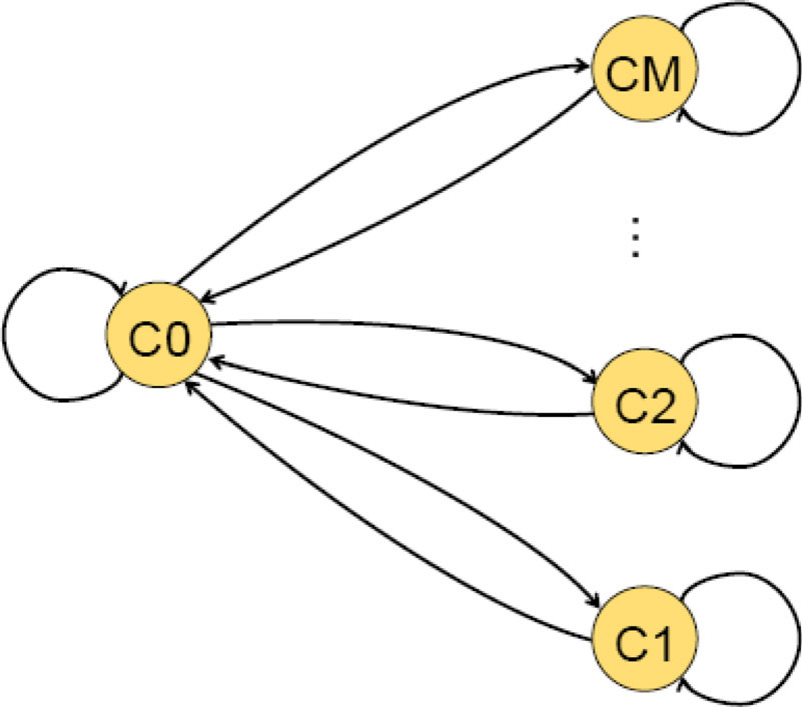
**Topology of HMM used in the algorithm for determining transcript boundaries and relative expression levels**.

**Figure 3:**
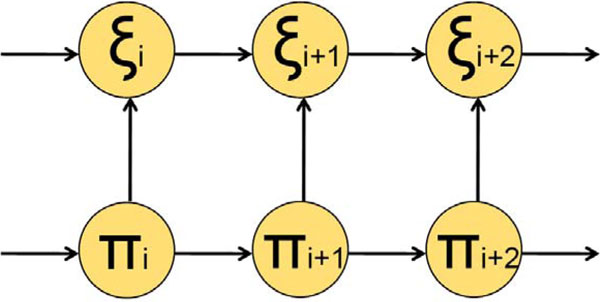
**Emission of an observed state in a current position depends on emission of the observed state in previous position**.

To infer transcript boundaries and expression levels from the noisy coverage data we have developed a Hidden Markov model (HMM) based algorithm (Figure 2). The coverage data were divided into bins corresponding to the observable states. The HMM hidden states include a zero state, C0, emitting zero coverage and *M *expression states, C1... CM, emitting positive coverage values ranging from C1 the lowest to CM the highest. The number of expression states, *M*, was chosen by increasing the number of expression states until the break point for the C1 bin decreased to a coverage ≤ 2; for analysis of the current set of RNA-Seq data we had M = 9. Notably, in this HMM an emission for a given position depends on the coverage count emitted in the previous position (Figure 3). Emission probabilities as well as transition probabilities were estimated during the model training stage (see below).

With HMM model and parameters in place we implemented an algorithm for inferring the most likely parse of the sequence into transcribed regions with constant expression levels (C0-C9). This algorithm was the Viterbi type algorithm for an HMM with the first order dependence between observed variables. Since the input mapped reads contained an assignment to a particular DNA strand, the algorithm was run twice (once for each strand) on the mapped RNA-Seq reads from RNA generated in the four cell growth conditions. The output of the algorithm was the predicted positions of transcription start sites (TSS) and transcription end sites (TES) as well as the expression levels for each predicted transcript.

### HMM Training

We used the NCBI curated gene annotation of *B. anthracis *str. 'Ames Ancestor' provided in the RefSeq record NC_007530 as well as the complement of genes predicted by GeneMarkS [1]. These two gene sets have a significant overlap.

We assumed that confidently identified protein-coding regions are *bona fide *transcribed regions. Thus we used these regions along with the mapped RNA-Seq reads as a training set for estimation the HMM parameters. To determine the probability of remaining in the zero expression state *C*0, *a*_*C*0-*C*0_, the length distribution of regions with zero expression was determined directly from the raw coverage data. The mean of the distribution *μ*_*zero *_was 56 nt. Then, assuming the geometric length distribution for non-transcribed regions, . Similarly, to assign transition probabilities for each of the expression states we used the estimated average length of an operon, *μ*_*operon *_= 5 kb. We then set the self-transition probabilities of these states to: . Finally, the probability of transition back to the zero state was: .

Emission probabilities for each hidden state were calculated from the coverage data as follows: i/ the whole range of possible coverage values was divided into 12 bins with break points chosen to have an equal number of genome positions fall into each bin; ii/ the average coverage of each annotated gene was calculated (with exclusion of 50 nt downstream from the 5' end); iii/ the range of values of the averaged coverage was divided into M bins (C1, ..., CM) with break points chosen to have an equal number of genes with non-zero expression in each bin; iv/ the emission counts were initialized with Laplace's pseudocounts; v/ for each position, the first-order dependence of the emission counts from the emission in the previous position was determined; vi) the emission counts were converted into emission probabilities.

### Finding a promoter motif in sequences upstream to TSS

To identify promoter-containing sequences, we first extracted 60 nt upstream of the predicted TSSs. It was required that each sequence meets the criteria: i/ the 5' UTR is between 10 and 200 nt in length; ii/ the 5' UTR is not part of any other predicted transcript; iii/ the most 5' gene in the transcript has a median coverage ≥ 5.

**Figure 4:**
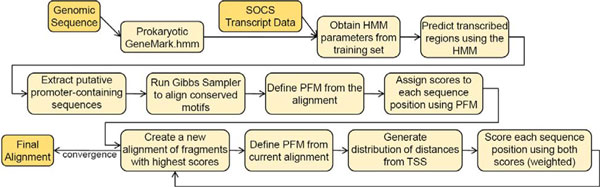
**A diagram for the iterative algorithm used for promoter motif finding**. Dark yellow boxes represent input/output.

To identify the -10 motifs we used a two step approach. First, we ran a standard Gibbs sampler algorithm [21] to determine the initial "-10" motif. Thus identified sequence fragments, the instances of the motif, were then used as a starting point for a modified Gibbs sampler with the scoring function  that takes into account the length distribution of spacers (sequences from -10 motif to the predicted TSS). Here *ω*_1 _and *ω*_2 _designate the weights of (i) the positional frequency term that depends on the -10 motif model P_*i*_(*α*) and the background model Q_*α *_as well as (ii) probability *P*(*d*) of the spacer length. A diagram of the iterative motif finding algorithm is shown in Figure 4. The algorithm runs until no motif sequences shift position from previous iteration to the next.

**Figure 5:**
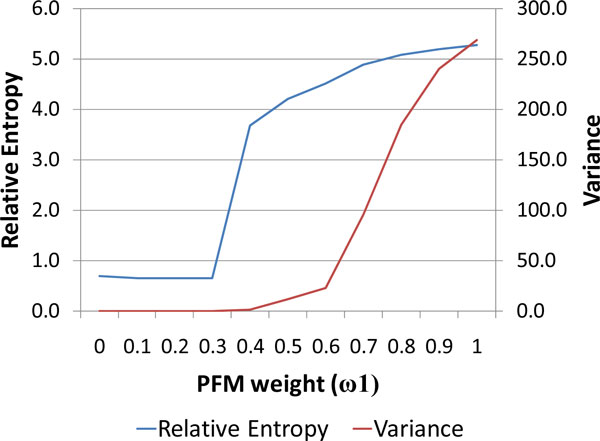
**Relative entropy of the promoter motif model and variance of the spacer length distribution are shown as functions of the combined score weight parameter *ω*_1_**.

Interestingly, both the relative entropy of the motif model and the variance of the length distribution *P*(*d*) have shown a Hill-function type dependence on the value of *ω*_1_(Figure 5). Here the sharp rise in relative entropy at *ω*_1 _= 0.3 indicates a percolation effect of instant accumulation of motif instances with similar (conserved) sequences due to the activation of positive feedback link formed by the multiple sequence alignment. At this point *ω*_1 _= 0.3 the balance in the score function S starts to shift to the first term of the equation that controls the quality of alignment. The growth of the variance at *ω*_1_= 0.6 indicates the point where the motif information part of the score becomes ultimately dominant in comparison with the spacer length distribution term. At this point the motif instances identified by the algorithm start to spread around and the preference to concentrate at an "optimal" distance from TSS is fading away. Value *ω*_1_= 0.6 was chosen to generate a model with high information (relative entropy) and a compact (low variance) length distribution of spacers.

### Transcription terminator sequences

For identification of transcription terminator sequences we used the TransTermHP program [3]. This algorithm locates rho independent terminator sequences by searching for specific secondary stem-loop structures. The software is available along with pre-computed predictions for a number of bacterial species, including *B. anthracis *str. 'Ames Ancestor'. We used transcription terminator predictions made by TransTermHP version 2.07; specifically 3407 "best after gene" predictions were used. TransTermHP provides scores for hairpin and tail structures that are related to efficiency of termination. For genes with 3' UTRs longer than 200 nt we have compared locations of transcription end sites (TES) predicted by the Viterbi algorithm with the locations of transcription terminators predicted by TransTermHP.

### Measures of translational efficiency

As a predictor of translational efficiency for an mRNA we introduced an average translation speed (ATS) defined as follows. Let frequencies of 61 codons in a reference gene set be s_i_, i = 1,2, ..., 61. Since evolutionary adaptation of the codon and anticodon (tRNA) populations is supposed to eliminate disproportions at a time of fast growth, we assume that the frequencies of tRNA in a cell are proportional to s_i _values. Before a cognate tRNA is admitted to the A site at a ribosome, a number of candidate tRNA are tried and rejected. We assume a Poisson process for interactions between a cognate tRNAs and the ribosome A site; thus, the average time needed for recruiting a cognate tRNA is proportional to 1/s_i_. For a gene with N codons and k_i _codons of each kind the average time of mRNA translation is T = ∑k_i_/s_i_. Then, for a given gene the average time of a codon translation is t = T/N = ∑(k_i_/N)/s_i_. Finally, with k_i_/N being a frequency of a codon i in the gene, designated as f_i_, we have t = ∑f_i_/s_i _and the average speed of translation of the gene is V = (∑f_i_/s_i_)^-1^. More accurate computation of the average speed of codon translation requires data on concentration of each mRNA, knowledge that has not been available until recently. In this study we use the RNA-Seq derived information on gene expression levels observed in *B. anthracis *(see below) to make correction in the s_i _values. Instead of s_i _defined as ∑*μ*^j^_i_/∑∑*μ*^j^_i _for each codon type i among the genes in the reference set, with *μ*^j^_i _being a count of codons of type i in gene j, we used the formula, S_i _= ∑w_j _*μ*^j^_i_/∑∑w_j _*μ*^j^_i _where w_j _is the expression level of gene j. Now the formula for V can be modified and we defined the value of ATS index, the average translation speed of a gene, ATS = (∑f_i_/S_i_)^-1^. For comparison, we also used the classic CAI measure defined by Sharp and Li [4].

### RBS scores

The GeneMarkS program as a part of gene prediction algorithm determines parameters of the RBS positional frequency model and the RBS spacer, a sequence between RBS and translation start site, length distribution. The program also computes a score of the RBS related to a predicted gene. The score is defined by the formula  similar in structure to the promoter score discussed above. Here *σ*_1 _and *σ*_2 _are the weights of the positional frequency term and spacer distribution terms (in computations we had *σ*_1 _= *σ*_2 _= 1);  is the set of positional nucleotide frequencies in the RBS model and *Q*_*x *_is the set of nucleotide frequencies in the background model.

## Results and discussion

### Comparison of annotated and predicted protein-coding genes with transcripts inferred from RNA-Seq mapped reads

We compiled a set of *B. anthracis *"candidate genes", by augmenting a set of genes annotated in RefSeq (NC_007530) by a few additional genes predicted by GeneMarkS. Each of the candidate genes could be verified by the RNA-Seq data. We have inferred the set of transcripts by running the HMM based algorithm twice (once for each strand) on the RNA-Seq coverage data obtained for the four cell growth conditions (see Methods). Each candidate gene was assigned to a transcript with which the gene shared the largest overlap. If there was at least one condition where the gene was predicted to be expressed to at least C2 level, then the gene was designated as an expressed one.

The predicted transcripts were used for assessment of the candidate gene calling accuracy. Each gene was classified as: i/ a gene both predicted by GeneMarkS and annotated in RefSeq (if predicted and annotated genes had the same 3' ends); ii/ a gene predicted but not annotated; iii/ a gene annotated but not predicted. Each gene in the three groups of candidate genes (Table 1) was counted as confirmed if it was covered by a transcript derived from the RNA-Seq data.

**Table 1:** Fraction of expressed genes among different categories. The fact of expression was inferred from the RNA-Seq data. *Predicted genes include pseudogenes.

	Total	Expressed	Fraction
Genes predicted and annotated	5144	4591	89.2%

Genes predicted but not annotated as genes*	517	421	81.4%

Genes annotated but not predicted	164	119	72.6%

### RNA expression as a necessary condition for the presence of a protein coding gene

It is quite straightforward to state that a gene is expressed based on observed RNA-Seq coverage, especially if the coverage level is high; however, there is less confidence in questioning an existence of a predicted/annotated gene based on observed low transcription levels (due to a noise in experiments and possibility of missing condition-specific gene expression).

**Figure 6:**
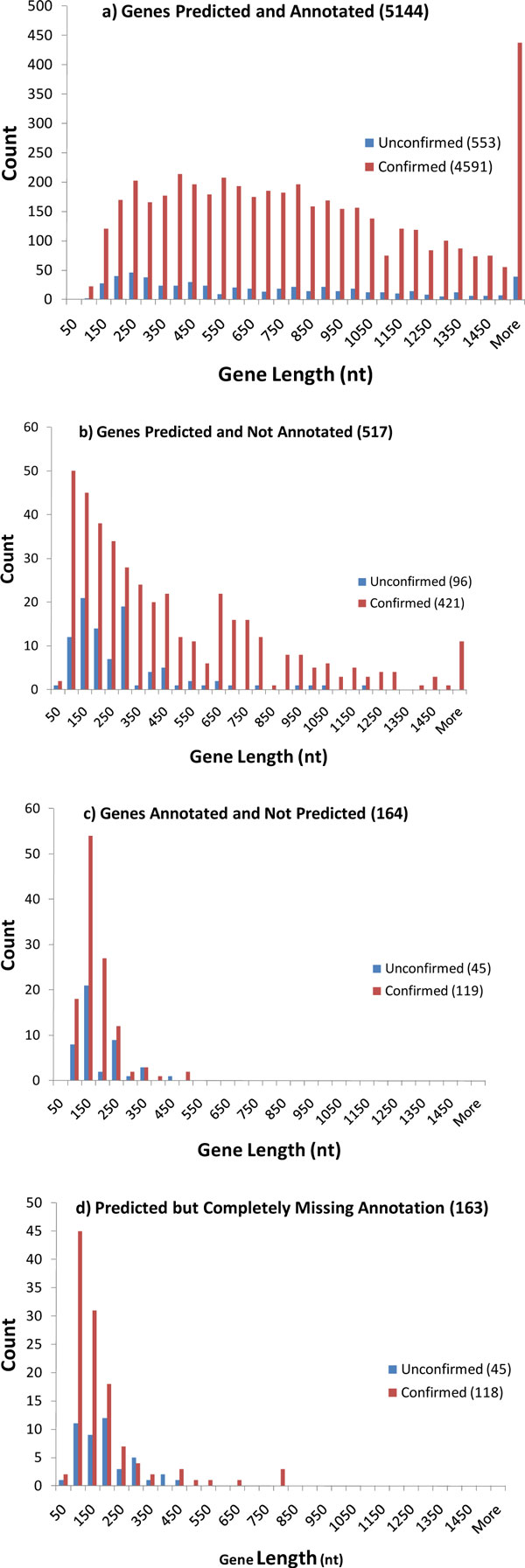
**Length distributions for four categories of *B. anthracis* genes.**. Genes are classified as confirmed or unconfirmed with regard to the inferred expression level.

For genes both annotated and predicted (Figure 6a) the distribution of the number of confirmed genes with respect to their length repeats the shape of the annotated gene length distribution. The RNA-Seq supporting evidence is quite uniform, though the genes of shorter length (≤ 350 nt) lack the transcript evidence more frequently.

**Figure 7:**
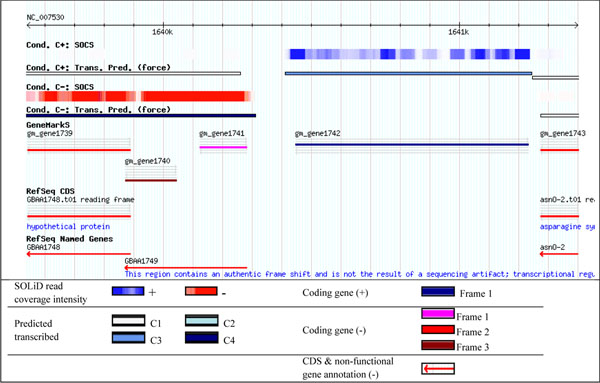
**An example of the genome browser view**. Positions 1639540-1641400 of the *B. anthracis *genomic sequence are displayed. Gene 1742 is predicted by GeneMarkS but not annotated, while genes 1740 and 1741 are predicted but annotated in RefSeq as a frameshift region; genes 1739 and 1743 are both predicted and annotated.

Next, there are 517 genes predicted by GeneMarkS but not annotated in RefSeq. Interestingly, 421 out of 517 predicted genes appear to be transcribed. To give an example we placed the RefSeq annotation and transcript coverage data together into the Gbrowse genome browser [22]. Figure 7 shows a segment of the *B. anthracis *genome between positions 1,639,540 - 1,641,400; one can see a gene with a length of about 800 nt in the forward strand predicted by GeneMarkS (with expression level C3 in the log-air, control growth conditions). This gene was predicted but not annotated.

Similar to the aforementioned pattern, the shorter genes are lacking the expression evidence more frequently than the longer genes (Figure 6b). Notably, the support by expression data of 421 newly predicted *B. anthracis *genes indicates that predicted protein coding regions are not likely to be artifacts of gene prediction. This discrepancy was largely resolved by taking into account not only annotated genes but also pseudogenes.

Notably, in 332 out of 517 loci where new genes were predicted by GeneMarkS the RefSeq annotates various classes of pseudogenes (Table 2, Figure 7). Still, RefSeq annotates neither gene nor a pseudogene in yet another 163 loci where GeneMarkS predicts other new genes (Table 2).

**Table 2:** Detailed categorization of new genes predicted by GeneMarkS. Note that the first five rows correspond to cases of frameshift or premature stop annotations and subtotal to 332 gene predictions.

The region contains an authentic frameshift and is not the result of a sequencing artifact	210
The region contains an authentic point mutation causing a premature stop and is not the result of a sequencing artifact	56

The region contains a gene with one or more premature stops or frameshifts and is not the result of a sequencing artifact	51

The region contains a pseudo gene one or more premature stops and is not the result of a sequencing artifact	10

The region contains a match to at least one other gene that is not full length and is not the result of a sequencing artifact	5

The region is annotated as rRNA	22

Not annotated	163

**Total**	**517**

We found that 118 of the 163 new genes (72.4%) are expressed at RNA level using the same set of criteria as above. Most of these non-annotated genes are relatively short (Figure 6d).

Further, we have considered 164 genes annotated but not predicted by GeneMarkS (Figure 6c). Almost all of these genes were ≤ 350 nt in length; many were supported by the transcriptome evidence (72.6%). Overall, the graphs for both "new" and "missing" genes (Figure 6bc) suggest that most of the likely erroneously predicted or annotated genes occur in the range of gene length ≤ 350 nt.

Does GeneMarkS identify all of the pseudogenes annotated by RefSeq? Only 11 loci in *B. anthracis *genome where RefSeq record annotates genes with frameshifts, premature stops, and/or pseudogene are lacking GeneMarkS predictions of protein-coding regions. This statistics of pseudogene recognition indicates that a protein-coding type of nucleotide ordering remains in a sequence (and is detected by GeneMarkS) for a long time since mutations made a gene to lose its function. Amazingly, the promoters frequently seem to remain active, thus recruiting RNA polymerase to generate transcripts for pseudogene regions.

### Identification of promoter sequences

**Figure 8:**
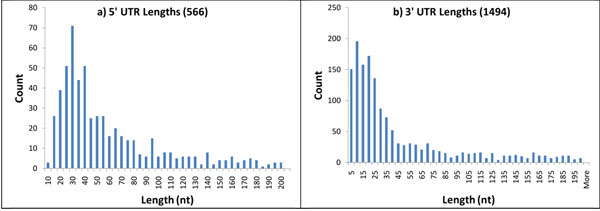
**Length distributions of 5' and 3' UTR**. **a.** TSSs were defined as 5' boundaries of transcripts identified by the Viterbi algorithm. **b**. Length distribution of predicted 3' UTR. TESs were defined as 3' boundaries of transcripts identified by the Viterbi algorithm.

We have chosen 566 *B. anthracis *genes whose 5' untranslated upstream regions satisfied the selection criteria (see Methods). The length distribution of the 5' UTRs for this set of 566 transcripts is shown in Figure 8a. The length of 5' UTRs is well conserved, with mean length 58 nt and 67% of the UTRs being ≤ 60 nt in length. Next, we selected 60 nt fragments upstream to RNA-Seq defined TSS locations. These fragments were expected to contain core RNA polymerase binding sites.

**Figure 9:**
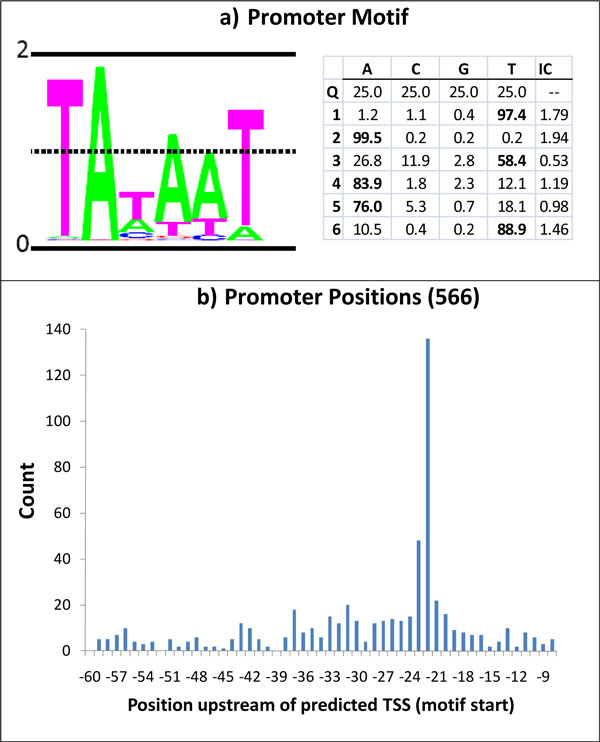
**Logo of the Pribnow  box motif. **a) Logo of Pribnow box motif detected within 60 nt upstream of predicted TSSs. b) Distribution of Pribnow box start positions relatively to predicted TSSs.

The iterative promoter motif refinement algorithm (see Methods) was applied to the set of 566 sequences. This multiple sequence alignment algorithm converged after 13 iterations and produced -10 region motif with a well-known TATAAT consensus (Figure 9a). The conservation of the hexamer motif is relatively high, having average information content (relative entropy) of 1.3 per position. Notably, almost all aligned fragments use consensus nucleotides T and A in positions one and two of the motif, respectively.

It is thought that in addition to the conservation of the -10 promoter motif, the distance from the -10 motif instances to starts of transcription is also well conserved. We found that on average in *B. anthracis *the start of the conserved hexamer is 22 nt upstream to a transcription start site (Figure 9b); with an average 16 nt long spacer between the end of the Pribnow box and TSS.

### Promoter score correlation with expression level of regulated genes

**Figure 10:**
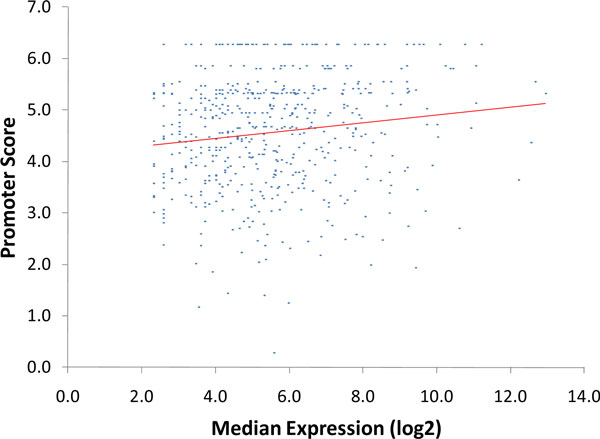
**Joint distribution of promoter scores and downstream gene expression levels**. Correlation coefficient is 0.14.

It is interesting to determine a relation between promoter strength and expression level of the downstream gene. Here we used a combined promoter score, which accounts for both the promoter motif sequence and the distance from motif to TSS. It is expected that strong promoters attract RNA polymerase more efficiently, initiate transcription more frequently and, thus, contribute to high gene expression. To check this hypothesis we plotted the median expression of 566 genes determined in the control growth condition against the score of upstream promoter (Figure 10). We found that there is indeed a positive correlation between gene expression level and the promoter score. The correlation coefficient is rather small 0.14. However, this score reflecting the sequence of the Pribnow box is arguably related to a basal gene expression; also, there are other important factors that influence gene expression, such as regulatory proteins.

### Characterization of terminator sequence elements

In order to elucidate yet another detail of the *B. anthracis *genome organization we analyzed the 3' UTR and terminator sequences. We considered 5334 *B. anthracis *genes with stop codons situated in the regions identified as transcribed by the Viterbi algorithm. In order to obtain reliable 3' UTRs and positions of transcription end sites (TESs), this set was further filtered (see Methods) to result in a set of 1494 3' UTR sequences. The 3' UTR length distribution (Figure 8b) has a mean of 43 nt; 54% of the 3' UTRs are ≤ 25 nt in length. The shorter average length of 3' UTR in comparison with 5' UTR, indicates that 5' UTRs provide a larger room for regulatory sequences at translation level including the RBS site.

**Figure 11:**
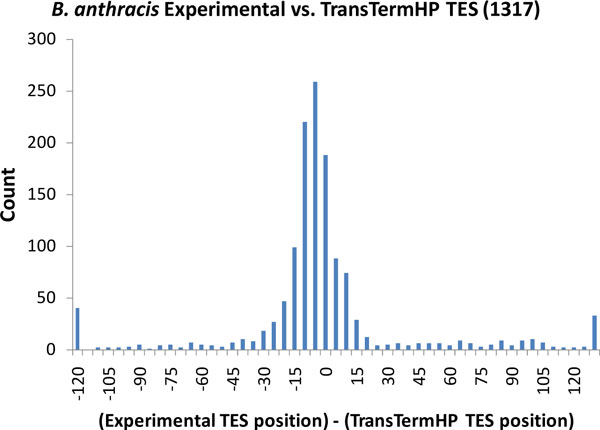
**Comparison of TES positions predicted by TransTermHP with ones defined from RNA-Seq data**. Negative values correspond to experimental TES locating upstream of the TransTermHP prediction. The relative positions were calculated for 1317 genes.

We compared the TES locations inferred from the mapped RNA-Seq reads with locations of transcription terminators ("middle" positions) predicted by the TransTermHP program [3]; it was done for 1317 genes possessing both types of predictions. We have shown that 65% of the TESs reside in -25 to +5 vicinity from the predicted terminators (Figure 11). This result indicated that the TransTermHP 'best after gene" predictions are quite accurate.

### Transcription terminator score correlation patterns

**Figure 12:**
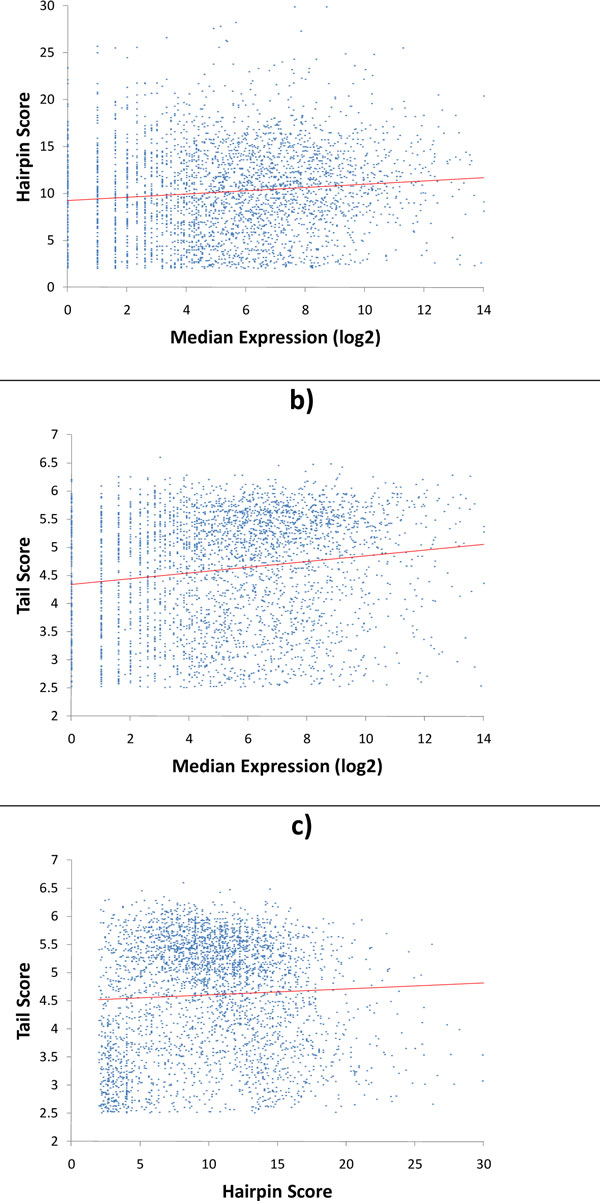
**Joint distributions of gene expression level and a) terminator hairpin and b) tail scores determined by TransTermHP.** Hairpin scores are multiplied by (-1). Correlation coefficients are 0.12 and 0.16, respectively. Joint distribution of hairpin and tail score c) has correlation coefficient 0.05.

Similar to the analysis of promoter scores, we attempted to find a correlation between the hairpin and tail scores computed by TransTermHP with the level of gene expression of the upstream gene (Figures 12ab). We have observed weak positive correlation between gene expression level and both types of scores with correlation coefficient of 0.16 for the tail score and 0.12 for the hairpin score. This finding suggests a trend towards "stronger" terminators for more highly expressed genes, perhaps because the production of such important genes should be more tightly regulated.

Having a set of both promoter and terminator scores, we checked for correlation between them for a set of genes that have both promoters and terminators, the set of single gene operons (Figure 12c). With a correlation coefficient of -0.05 we conclude that promoter and terminator scores in single gene operons are essentially not correlated.

### Comparison of RNA-Seq operon mapping with OperonDB predictions

**Figure 13:**
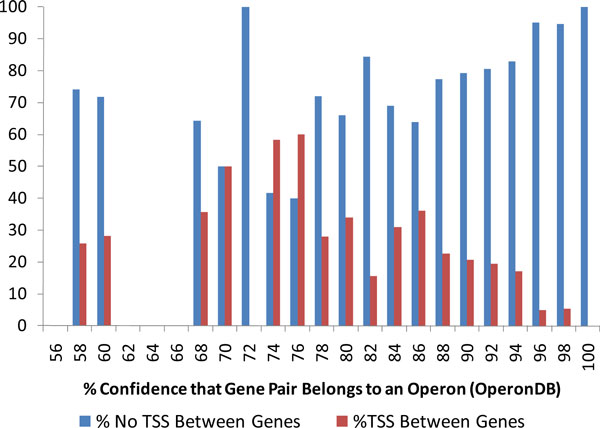
**Analysis of compatibility of the OperonDB predicted operon topology with locations of RNA-Seq inferred TSS site**. Confidence levels for adjacent genes to belong to the same operon were generated by OperonDB. For each confidence level the percent of cases when a TSS was predicted or not predicted between the two genes was calculated (for control growth data).

Transcripts inferred from mapped RNA-Seq reads determined extents of *B. anthracis *operons and delineate pairs of adjacent genes residing in the same operon. A computational tool for predicting operons in prokaryotic genomes, OperonDB, was developed earlier [2]. OperonDB defines a confidence level (%) for a pair of adjacent genes (located in the same strand) that the pair belongs to the same operon. To elucidate relationship between the OperonDB analysis and the transcript map predicted from raw RNA-Seq reads we determined for all gene pairs with given OperonDB defined confidence to belong to one operon the frequencies of two complementary events when RNA-Seq inferred TSS is present or absent between the two genes (Figure 13). Here we see that for the confidence level 75% and higher, the frequency of gene pairs with TSS between them (predicted from RNA-Seq) consistently goes down while the frequency of gene pairs with an evidence of TSS absence is steadily increasing. On the other hand, the Operon DB predictions with confidence from 50% to 75% are much less conclusive as could be expected.

### Relations of sequence determinants of translation efficiency with gene expression levels

The values of codon adaptation index and average translation speed for a given gene depend on the model parameters derived from codon frequencies in a selected reference set of genes. In the original paper Sharp and Li [4] used 27 *Escherichia coli *genes with experimentally demonstrated high expression. Obviously, orthologs of these genes in *B. anthracis *could make a reference set for computing CAI values for *B. anthracis *genes. However, several genes in the 27 strong set of *E. coli *genes do not have orthologs in *B. anthracis*. Therefore, we have added several ribosomal protein genes with the same total length, 1555 codons, to make up for the missing genes (Additional file 1, Supplementary Table 1). Interestingly, codons with highest frequencies (optimal codons) in the groups of synonymous codons, are not the same in the reference set of highly expressed genes and in the whole complement of *B. anthracis *genes (Additional file 2).

Gene expression data delivered by mapped RNA-Seq reads allows for ranking genes by expression levels. For the sake of comparison, we have increased the size of the reference set to 100 genes, with 48 of these genes coding for ribosomal proteins (Additional file 1 Supplementary Table 2). A comparison of codon frequencies in the whole complement of genes and in the 100 most highly expressed genes (under Control condition) shows (Additional file 2) that seven synonymous groups have different optimal codons. The list of optimal codons is interesting to compare with the list of tRNA genes (Additional file 2). The anti-codon identities of the *B. anthracis *tRNA genes could be inferred either from the RefSeq annotation (NC_007530) or by using the tRNA gene finding program, tRNASCAN-SE [23]. Notably, 34 out of 61 codons do not have tRNAs with exactly matching anti-codon, which is in agreement with the "wobble hypothesis" [24], suggesting that some tRNA species could pair with more than one codon. In 6 out of 18 cases the optimal codon in 100 highly expressed genes does not match the exact tRNA species present in the *B. anthracis *cell; the optimal codon in the whole gene complement does not match the exact tRNA species in 9 out of 18 cases.

### Relation of average translation speed and RBS score with gene expression level

**Figure 14:**
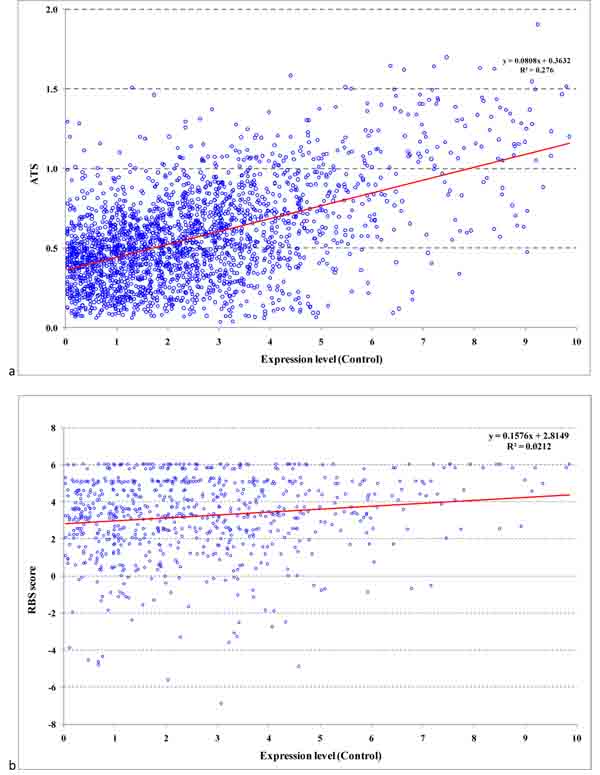
**a) Joint distribution of 100 gene expression-weighted ATS index and gene expression level; b) joint distribution of RBS score and gene expression level**.

**Figure 15:**
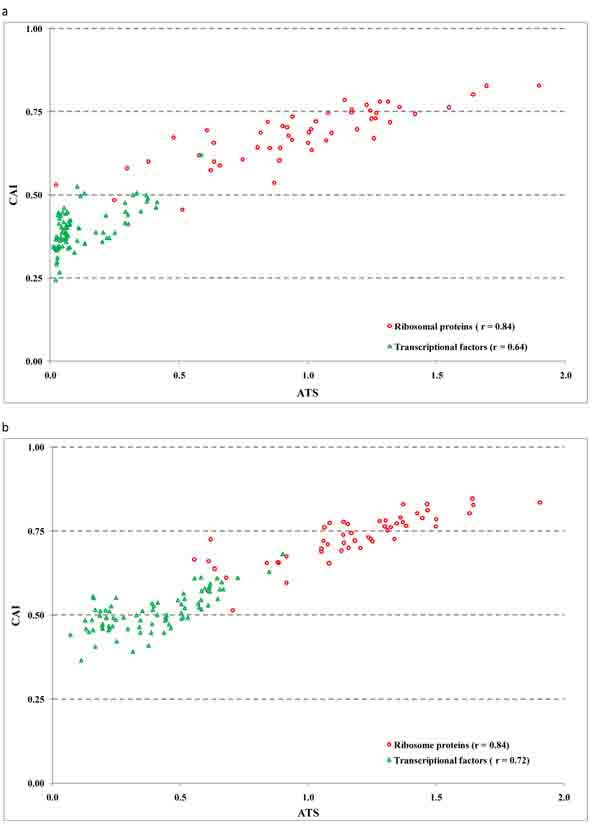
**Joint distribution of ATS (weighted) and CAI values for genes of ribosomal proteins and transcription factors. **ATS and CAI were defined based on the reference set of a) *B. anthracis* genes of 37 proteins homologous to highly expressed *E. coli *proteins (including those selected by Sharp 1986); b) 100 most highly expressed *B. anthracis* genes as inferred from the RNA-Seq data.

It was inferred from the mapped RNA-Seq reads that under control growth condition 2,375 *B. anthracis *genes are expressed and have an average coverage by transcript reads larger than 1 (each gene position is covered by a RNA-Seq read more than once on average). We have determined the weighted ATS value for each gene using the 100 most highly expressed genes as a reference set for computation (see Methods) and plotted ATS as function of (log2) gene expression level (Figure 14a). We found that ATS correlates with gene expression level with correlation coefficient equal to 0.525. Also we determined the values of codon adaptation index, CAI, for each gene using either 37 or 100 highly expressed genes as a reference set. Similarly, we plotted CAI values, as a function of gene expression level. (Additional file 3, Supplementary Figures 1a,b). These figures show almost identical behavior of CAI with respect to a choice of the reference set. We compared the values of CAI and ATS for sets of ribosomal protein genes and genes of transcription factors (Additional file 1, Supplementary Tables 3, 4) for two reference sets: 37 and 100 highly expressed genes (Figure 15a,b). One can see that computation of CAI and weighted ATS based on the smaller set of 37 genes provides better separation of the two groups of genes with high and low expression levels.

We have also revisited the issue of relations of RBS scores and expression levels of downstream genes. To avoid genes whose translation might start by a ribosome that just finished translation of the upstream gene situated in close proximity and, was not fully disassembled, we selected genes preceded by non-coding regions longer than 100 nt. From this set we further selected a subset with average coverage by RNA-Seq reads larger than 1, a total of 748 genes. In contrast with earlier observation of no correlation between the RBS score and gene expression level [7] we did observe a weak but significant correlation (Figure 14b) with correlation coefficient 0.158. This result means that there is a trend for genes with higher expression to have stronger RBS sites. This trend could be expected as genes expressed at high level need to be tightly regulated at all levels including the translation level. We zoom in on genes with likely *de novo *ribosome binding and assembling and filter out genes where RBS may not play a decisive role in the process of translation initiation.

### Correlation of gene expression levels for adjacent genes

**Figure 16:**
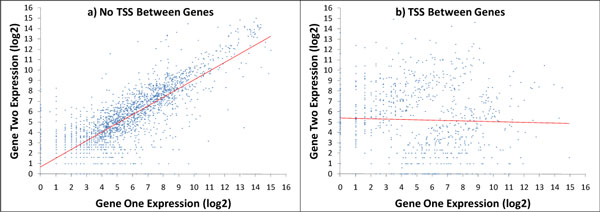
**Joint distributions of expression levels of pairs of adjacent genes located in the same strand a) with no TSS predicted between the pair of genes, and b) with TSS predicted between the pair of genes**. Correlation coefficients were 0.85 and -0.03, respectively.

Finally, correlation of gene expression levels of adjacent genes is expected if two genes belong to the same operon. Predicted TSS and TES positions delineate the *B. anthracis *operons, however, we anticipate that there will be a certain fraction of false positive TSSs and TESs due to the presence of errors in obtaining transcript reads and in their mapping. Also, some true TSS and TES could be missed. Figure 16a shows the gene expression levels for gene pairs that are presumably situated in one operon, i.e. in between a pair of TSS and TES predicted by the algorithm. Figure 16b shows gene expression levels for pairs of genes divided by transcript borders, a pair of TES and TSS. These two figures clearly illustrate correlation of gene expression levels for the gene pairs situated in the same transcript (r = 0.85) and absence of correlation for gene pairs divided between two adjacent transcripts (r = -0.03).

## Concluding remarks

Overall, the findings reported in this paper are as follows. We have shown, in the case of bacteria *B. anthracis*, that noisy data on mapped RNA-Seq reads can be used in an HMM based algorithm inferring transcribed and non-transcribed regions. We have shown that thus determined transcribed regions provide evidence for transcription for majority of already annotated active genes, for some mutated dysfunctional genes, pseudogenes, as well as for some genes predicted by an *ab initio *gene finder but not annotated yet. It was shown that adjacent genes located inside predicted transcripts have strong correlation in expression levels, while adjacent genes residing in different transcripts do not exhibit a correlation at all. We have shown that the OperonDB predictions of pairs of genes situated in the same operon are in agreement with the evidence derived from mapped RNA-Seq reads.

It was shown that promoter sites identified upstream to the predicted TSS have scores that correlate with gene expression level of downstream genes. Also, we found a weak correlation of the gene expression level with the scores of hairpin and tail of the downstream transcription terminator. We have shown that the locations of sites of transcription termination predicted by TransTermHP are in good agreement with the TES sites inferred from RNA-Seq data. We have shown that the new ATS index, the average translation speed of a gene, as well as CAI correlate with gene expression level. Also, contrary to what was thought before, we found a correlation of the score of an RBS site with gene expression level of the downstream gene for genes that appear to be the first genes in operons.

## Competing interests

The authors declare that they have no competing interests.

## Authors' contributions

MB and NB conceived the project and design. KDP prepared the RNA-seq data. JM and WZ implemented the algorithms, performed the computational experiments, and analyzed the results. JM, NB and MB wrote the paper. All authors read and approved the document.

## Supplementary Material

Additional file 1 **Joint distribution of gene expression levels and CAI values.** CAI was calculated using a) 37 proteins homologous to highly expressed *E. coli *proteins (including those selected by Sharp 1987); b) 100 most highly expressed genes inferred from the RNA-Seq data. There is no obvious advantage in using a larger set of genes with high expression. Click here for file

Additional file 2 **Codon frequencies in the genome wide set of genes and its subsets along with frequencies of cognate tRNA genes for each codon.** The codon frequencies were calculated from the three sets of coding sequences, namely the whole genome, the 100 most highly expressed genes as observed from the read coverage data and the 37 homologs to the proteins used by Sharp et. al (1987). The "Weighted 100 genes" column shows the frequencies of codons adjusted by weights, expression levels of the 100 genes as determined from read coverage data. The 95 tRNA genes shown in "tRNA genes" column were assigned to codons by tRNAscan-SE. Codon frequencies are normalized to 1000. Numbers in bold font indicate the maximum frequencies/counts in a synonymous group. Click here for file

Additional file 3 Supplementary tables. Click here for file
