# Supplementary material for: Bacillus anthracis genome organization in light of whole transcriptome sequencing
Source: BMC Bioinformatics. 2010 Apr 29;11(Suppl 3):S10. doi: 10.1186/1471-2105-11-S3-S10 (PMC2863060; doi:10.1186/1471-2105-11-S3-S10)
Supplement: Additional file 1 — Joint distribution of gene expression levels and CAI values. CAI was calculated using a) 37 proteins homologous to highly expressed E. coli proteins (including those selected by Sharp 1987); b) 100 most highly expressed genes inferred from the RNA-Seq data. There is no obvious advantage in using a larger set of genes with high expression. [file 1471-2105-11-S3-S10-S1.doc]

**Supplementary Table 1**. A list of 37 genes used to calculate codon adaptation index. Out of these 37 genes 23 are homologous to the *E. coli* genes used by Sharp et al. (1987). Other 14 genes were added to make up to the same number of codons as in original set of Sharp et al. (1987). The table is sorted in descending order of the expression level. The first 31 genes are in the data set of the 100 most highly expressed genes (Supplementary Table 2). *The gene #37 homologous to an *E. coli* gene in the Sharp et al. (1987) set has a very low level of expression (0.2). It is likely to be an artifact which still should not bias computed ATS value, as in computations the codon counts from gene #37 will be added as they are.

| **Index** | **Strand** | **Left end** | **Right end** | **Length(nt)** | **Function** | **Expression level, log2 (Control)** |
| --- | --- | --- | --- | --- | --- | --- |
| 1 | + | 119376 | 120563 | 1188 | elongation factor Tu | 9.9 |
| 2 | - | 5214543 | 5214833 | 291 | 30S ribosomal protein S6 | 9.7 |
| 3 | - | 3621884 | 3622153 | 270 | 30S ribosomal protein S15 | 9.3 |
| 4 | + | 107065 | 107424 | 360 | 50S ribosomal protein L7/L12 | 9.2 |
| 5 | + | 128969 | 129469 | 501 | 30S ribosomal protein S5 | 9.2 |
| 6 | + | 106497 | 106997 | 501 | 50S ribosomal protein L10 | 9.2 |
| 7 | + | 116502 | 116972 | 471 | 30S ribosomal protein S7 | 9.1 |
| 8 | + | 133741 | 134130 | 390 | 30S ribosomal protein S11 | 9.1 |
| 9 | + | 116050 | 116472 | 423 | 30S ribosomal protein S12 | 9.0 |
| 10 | + | 117180 | 119258 | 2079 | elongation factor G | 8.9 |
| 11 | - | 4122609 | 4122782 | 174 | 30S ribosomal protein S21 | 8.8 |
| 12 | + | 121305 | 121937 | 633 | 50S ribosomal protein L3 | 8.8 |
| 13 | + | 120962 | 121270 | 309 | 30S ribosomal protein S10 | 8.8 |
| 14 | + | 124437 | 125096 | 660 | 30S ribosomal protein S3 | 8.7 |
| 15 | + | 127583 | 127981 | 399 | 30S ribosomal protein S8 | 8.6 |
| 16 | + | 123796 | 124074 | 279 | 30S ribosomal protein S19 | 8.6 |
| 17 | + | 125743 | 126006 | 264 | 30S ribosomal protein S17 | 8.5 |
| 18 | + | 104969 | 105394 | 426 | 50S ribosomal protein L11 | 8.5 |
| 19 | - | 3642203 | 3642904 | 702 | 30S ribosomal protein S2 | 8.2 |
| 20 | - | 5227127 | 5227261 | 135 | 50S ribosomal protein L34 | 8.2 |
| 21 | + | 133351 | 133716 | 366 | 30S ribosomal protein S13 | 8.1 |
| 22 | + | 135291 | 135653 | 363 | 50S ribosomal protein L17 | 8.1 |
| 23 | - | 4082555 | 4082704 | 258 | 50S ribosomal protein L33 | 8.0 |
| 24 | - | 5213716 | 5213949 | 234 | 30S ribosomal protein S18 | 7.8 |
| 25 | + | 4459692 | 4460294 | 603 | 30S ribosomal protein S4 | 7.7 |
| 26 | - | 4082555 | 4082704 | 150 | 50S ribosomal protein L33 | 7.6 |
| 27 | + | 127368 | 127553 | 186 | 30S ribosomal protein S14 | 7.6 |
| 28 | + | 105572 | 106264 | 693 | 50S ribosomal protein L1 | 7.2 |
| 29 | - | 3641212 | 3642099 | 888 | elongation factor Ts | 7.1 |
| 30 | - | 4127654 | 4129489 | 1836 | molecular chaperone DnaK | 7.0 |
| 31 | - | 3658378 | 3658650 | 273 | 30S ribosomal protein S16 | 6.9 |
| 32 | + | 4136729 | 4136986 | 258 | 30S ribosomal protein S20 | 6.4 |
| 33 | + | 3674546 | 3674734 | 189 | 50S ribosomal protein L28 | 4.1 |
| 34 | + | 103778 | 103924 | 147 | 50S ribosomal protein L33 | 3.9 |
| 35 | + | 1436661 | 1437809 | 1149 | 30S ribosomal protein S1 | 3.8 |
| 36 | - | 3590395 | 3591753 | 1359 | recombinase A | 3.4 |
| 37 | - | 4153358 | 4153507 | 150 | 50S ribosomal protein L33* | 0.2 |

**Supplementary Table 2**. The 100 most highly expressed *Bacillus anthracis* genes under “Control” growth condition as determined from read coverage data. The list is sorted in descending order of the expression level.

| **Index** | **Strand** | **Left end** | **Right end** | **Length(nt)** | **Function** | **Expression level, log2 (Control)** |
| --- | --- | --- | --- | --- | --- | --- |
| 1 | + | 119376 | 120563 | 1188 | elongation factor Tu | 9.9 |
| 2 | - | 3299573 | 3299773 | 201 | cold shock protein CspB | 9.8 |
| 3 | - | 5214543 | 5214833 | 291 | 30S ribosomal protein S6 | 9.7 |
| 4 | + | 126795 | 127334 | 540 | 50S ribosomal protein L5 | 9.5 |
| 5 | - | 4251123 | 4251413 | 291 | 50S ribosomal protein L27 | 9.5 |
| 6 | - | 3621884 | 3622153 | 270 | 30S ribosomal protein S15 | 9.3 |
| 7 | + | 107065 | 107424 | 360 | 50S ribosomal protein L7/L12 | 9.2 |
| 8 | + | 128969 | 129469 | 501 | 30S ribosomal protein S5 | 9.2 |
| 9 | + | 106497 | 106997 | 501 | 50S ribosomal protein L10 | 9.2 |
| 10 | + | 116502 | 116972 | 471 | 30S ribosomal protein S7 | 9.1 |
| 11 | + | 896749 | 899193 | 2445 | s-layer protein sap | 9.1 |
| 12 | + | 133741 | 134130 | 390 | 30S ribosomal protein S11 | 9.1 |
| 13 | + | 130139 | 131440 | 1302 | preprotein translocase subunit SecY | 9.0 |
| 14 | + | 131497 | 132147 | 651 | adenylate kinase | 9.0 |
| 15 | + | 128585 | 128947 | 363 | 50S ribosomal protein L18 | 9.0 |
| 16 | + | 116050 | 116472 | 423 | 30S ribosomal protein S12 | 9.0 |
| 17 | + | 129483 | 129665 | 183 | 50S ribosomal protein L30 | 9.0 |
| 18 | + | 117180 | 119258 | 2079 | elongation factor G | 8.9 |
| 19 | - | 1606994 | 1607857 | 864 | flagellin | 8.9 |
| 20 | + | 132962 | 133180 | 219 | translation initiation factor IF-1 | 8.8 |
| 21 | - | 4122609 | 4122782 | 174 | 30S ribosomal protein S21 | 8.8 |
| 22 | + | 121305 | 121937 | 633 | 50S ribosomal protein L3 | 8.8 |
| 23 | + | 120962 | 121270 | 309 | 30S ribosomal protein S10 | 8.8 |
| 24 | + | 122905 | 123735 | 831 | 50S ribosomal protein L2 | 8.8 |
| 25 | + | 126457 | 126768 | 312 | 50S ribosomal protein L24 | 8.8 |
| 26 | + | 134311 | 135255 | 945 | DNA-directed RNA polymerase subunit alpha | 8.7 |
| 27 | + | 124437 | 125096 | 660 | 30S ribosomal protein S3 | 8.7 |
| 28 | + | 127583 | 127981 | 399 | 30S ribosomal protein S8 | 8.6 |
| 29 | + | 123796 | 124074 | 279 | 30S ribosomal protein S19 | 8.6 |
| 30 | + | 124092 | 124433 | 342 | 50S ribosomal protein L22 | 8.6 |
| 31 | + | 125743 | 126006 | 264 | 30S ribosomal protein S17 | 8.5 |
| 32 | + | 104969 | 105394 | 426 | 50S ribosomal protein L11 | 8.5 |
| 33 | - | 4122150 | 4122593 | 444 | gatb/yqey domain-containing protein | 8.5 |
| 34 | + | 122586 | 122876 | 291 | 50S ribosomal protein L23 | 8.5 |
| 35 | + | 125098 | 125532 | 435 | 50S ribosomal protein L16 | 8.5 |
| 36 | - | 4863474 | 4864478 | 1005 | glyceraldehyde-3-phosphate dehydrogenase | 8.4 |
| 37 | - | 4636892 | 4637092 | 201 | cold shock protein CspD | 8.4 |
| 38 | + | 133216 | 133329 | 114 | 50S ribosomal protein L36 | 8.4 |
| 39 | + | 126050 | 126418 | 369 | 50S ribosomal protein L14 | 8.3 |
| 40 | + | 121963 | 122586 | 624 | 50S ribosomal protein L4 | 8.3 |
| 41 | - | 3666095 | 3666328 | 234 | acyl carrier protein | 8.2 |
| 42 | - | 3642203 | 3642904 | 702 | 30S ribosomal protein S2 | 8.2 |
| 43 | - | 5227127 | 5227261 | 135 | 50S ribosomal protein L34 | 8.2 |
| 44 | + | 128014 | 128553 | 540 | 50S ribosomal protein L6 | 8.2 |
| 45 | + | 133351 | 133716 | 366 | 30S ribosomal protein S13 | 8.1 |
| 46 | - | 4251773 | 4252081 | 309 | 50S ribosomal protein L21 | 8.1 |
| 47 | + | 135291 | 135653 | 363 | 50S ribosomal protein L17 | 8.1 |
| 48 | + | 129699 | 130139 | 441 | 50S ribosomal protein L15 | 8.0 |
| 49 | - | 3744933 | 3745106 | 174 | 50S ribosomal protein L32 | 7.9 |
| 50 | - | 5213995 | 5214516 | 522 | single-stranded DNA-binding protein | 7.9 |
| 51 | - | 4648464 | 4649408 | 945 | L-lactate dehydrogenase | 7.8 |
| 52 | - | 5058593 | 5058838 | 246 | 50S ribosomal protein L31 type B | 7.8 |
| 53 | - | 5213716 | 5213949 | 234 | 30S ribosomal protein S18 | 7.8 |
| 54 | + | 108391 | 111924 | 3534 | DNA-directed RNA polymerase subunit beta | 7.7 |
| 55 | + | 125522 | 125722 | 201 | 50S ribosomal protein L29 | 7.7 |
| 56 | + | 132147 | 132893 | 747 | methionine aminopeptidase | 7.7 |
| 57 | + | 258543 | 258827 | 285 | co-chaperonin GroES | 7.7 |
| 58 | + | 4459692 | 4460294 | 603 | 30S ribosomal protein S4 | 7.7 |
| 59 | - | 1544452 | 1544649 | 198 | cold shock protein CspB | 7.6 |
| 60 | - | 4082555 | 4082704 | 150 | 50S ribosomal protein L33 | 7.6 |
| 61 | + | 127368 | 127553 | 186 | 30S ribosomal protein S14 | 7.6 |
| 62 | + | 1447230 | 1447502 | 273 | DNA-binding protein HU | 7.5 |
| 63 | - | 5037510 | 5037911 | 402 | F0F1 ATP synthase subunit epsilon | 7.5 |
| 64 | + | 111962 | 115573 | 3612 | DNA-directed RNA polymerase subunit beta' | 7.5 |
| 65 | + | 3530610 | 3530882 | 273 | DNA-binding protein HU | 7.5 |
| 66 | + | 226255 | 226362 | 108 | hypothetical protein | 7.4 |
| 67 | - | 4911264 | 4911806 | 543 | ribosomal subunit interface protein | 7.4 |
| 68 | + | 2047217 | 2047489 | 273 | hypothetical protein | 7.4 |
| 69 | - | 4858510 | 4859805 | 1296 | phosphopyruvate hydratase | 7.3 |
| 70 | + | 258866 | 260500 | 1635 | chaperonin GroEL | 7.3 |
| 71 | - | 4380540 | 4380740 | 201 | 50S ribosomal protein L35 | 7.3 |
| 72 | - | 4862150 | 4863334 | 1185 | phosphoglycerate kinase | 7.3 |
| 73 | - | 4861362 | 4862117 | 756 | triosephosphate isomerase | 7.2 |
| 74 | - | 4859836 | 4861365 | 1530 | phosphoglyceromutase | 7.2 |
| 75 | + | 105572 | 106264 | 693 | 50S ribosomal protein L1 | 7.2 |
| 76 | - | 3830293 | 3831270 | 978 | pyruvate dehydrogenase complex E1 component, beta subunit | 7.2 |
| 77 | - | 3658136 | 3658363 | 228 | kh domain-containing protein | 7.2 |
| 78 | + | 139149 | 139586 | 438 | 50S ribosomal protein L13 | 7.2 |
| 79 | - | 3641212 | 3642099 | 888 | elongation factor Ts | 7.1 |
| 80 | - | 4251417 | 4251707 | 291 | hypothetical protein | 7.1 |
| 81 | + | 503601 | 505850 | 2250 | formate acetyltransferase | 7.1 |
| 82 | + | 505920 | 506651 | 732 | pyruvate formate-lyase-activating enzyme | 7.1 |
| 83 | + | 1838333 | 1839595 | 1263 | NLP/P60 family protein | 7.1 |
| 84 | - | 4093878 | 4094489 | 612 | superoxide dismutase, Mn | 7.0 |
| 85 | - | 4127654 | 4129489 | 1836 | molecular chaperone DnaK | 7.0 |
| 86 | - | 3831274 | 3832389 | 1116 | pyruvate dehydrogenase complex E1 component, alpha subunit | 7.0 |
| 87 | - | 3656275 | 3656619 | 345 | 50S ribosomal protein L19 | 6.9 |
| 88 | - | 3658378 | 3658650 | 273 | 30S ribosomal protein S16 | 6.9 |
| 89 | + | 226405 | 226515 | 111 | hypothetical protein | 6.9 |
| 90 | - | 3828941 | 3830200 | 1260 | branched-chain alpha-keto acid dehydrogenase subunit E2 | 6.9 |
| 91 | - | 3901931 | 3903643 | 1713 | phosphoenolpyruvate-protein phosphotransferase | 6.9 |
| 92 | - | 5037932 | 5039341 | 1410 | F0F1 ATP synthase subunit beta | 6.9 |
| 93 | - | 5063560 | 5064417 | 858 | fructose-bisphosphate aldolase | 6.9 |
| 94 | + | 120795 | 120962 | 168 | hypothetical protein | 6.8 |
| 95 | - | 2690701 | 2690793 | 93 | hypothetical protein | 6.8 |
| 96 | - | 722230 | 723105 | 876 | quinol oxidase, subunit II | 6.7 |
| 97 | - | 4021331 | 4021723 | 393 | hypothetical protein | 6.7 |
| 98 | - | 355897 | 356460 | 564 | alkyl hydroperoxide reductase subunit C | 6.7 |
| 99 | - | 3827523 | 3828935 | 1413 | dihydrolipoamide dehydrogenase | 6.7 |
| 100 | - | 5040657 | 5042165 | 1509 | F0F1 ATP synthase subunit alpha | 6.7 |

**Supplementary Table 3**. A list of 52 *Bacillus anthracis* ribosomal protein genes. The expression level under “Control” growth condition was determined from read coverage data. The list is sorted in descending order of the expression level.

| **Index** | **Strand** | **Left end** | **Right end** | **Length (nt)** | **Function** | **Expression level, log2(Control)** |
| --- | --- | --- | --- | --- | --- | --- |
| 1 | - | 5214543 | 5214833 | 291 | 30S ribosomal protein S6 | 9.7 |
| 2 | + | 126795 | 127334 | 540 | 50S ribosomal protein L5 | 9.5 |
| 3 | - | 4251123 | 4251413 | 291 | 50S ribosomal protein L27 | 9.5 |
| 4 | - | 3621884 | 3622153 | 270 | 30S ribosomal protein S15 | 9.3 |
| 5 | + | 107065 | 107424 | 360 | 50S ribosomal protein L7/L12 | 9.2 |
| 6 | + | 128969 | 129469 | 501 | 30S ribosomal protein S5 | 9.2 |
| 7 | + | 106497 | 106997 | 501 | 50S ribosomal protein L10 | 9.2 |
| 8 | + | 116502 | 116972 | 471 | 30S ribosomal protein S7 | 9.1 |
| 9 | + | 133741 | 134130 | 390 | 30S ribosomal protein S11 | 9.1 |
| 10 | + | 128585 | 128947 | 363 | 50S ribosomal protein L18 | 9.0 |
| 11 | + | 116050 | 116472 | 423 | 30S ribosomal protein S12 | 9.0 |
| 12 | + | 129483 | 129665 | 183 | 50S ribosomal protein L30 | 9.0 |
| 13 | - | 4122609 | 4122782 | 174 | 30S ribosomal protein S21 | 8.8 |
| 14 | + | 121305 | 121937 | 633 | 50S ribosomal protein L3 | 8.8 |
| 15 | + | 120962 | 121270 | 309 | 30S ribosomal protein S10 | 8.8 |
| 16 | + | 122905 | 123735 | 831 | 50S ribosomal protein L2 | 8.8 |
| 17 | + | 126457 | 126768 | 312 | 50S ribosomal protein L24 | 8.8 |
| 18 | + | 124437 | 125096 | 660 | 30S ribosomal protein S3 | 8.7 |
| 19 | + | 127583 | 127981 | 399 | 30S ribosomal protein S8 | 8.6 |
| 20 | + | 123796 | 124074 | 279 | 30S ribosomal protein S19 | 8.6 |
| 21 | + | 124092 | 124433 | 342 | 50S ribosomal protein L22 | 8.6 |
| 22 | + | 125743 | 126006 | 264 | 30S ribosomal protein S17 | 8.5 |
| 23 | + | 104969 | 105394 | 426 | 50S ribosomal protein L11 | 8.5 |
| 24 | + | 122586 | 122876 | 291 | 50S ribosomal protein L23 | 8.5 |
| 25 | + | 125098 | 125532 | 435 | 50S ribosomal protein L16 | 8.5 |
| 26 | + | 133216 | 133329 | 114 | 50S ribosomal protein L36 | 8.4 |
| 27 | + | 126050 | 126418 | 369 | 50S ribosomal protein L14 | 8.3 |
| 28 | + | 121963 | 122586 | 624 | 50S ribosomal protein L4 | 8.3 |
| 29 | - | 3642203 | 3642904 | 702 | 30S ribosomal protein S2 | 8.2 |
| 30 | - | 5227127 | 5227261 | 135 | 50S ribosomal protein L34 | 8.2 |
| 31 | + | 128014 | 128553 | 540 | 50S ribosomal protein L6 | 8.2 |
| 32 | + | 133351 | 133716 | 366 | 30S ribosomal protein S13 | 8.1 |
| 33 | - | 4251773 | 4252081 | 309 | 50S ribosomal protein L21 | 8.1 |
| 34 | + | 135291 | 135653 | 363 | 50S ribosomal protein L17 | 8.1 |
| 35 | + | 129699 | 130139 | 441 | 50S ribosomal protein L15 | 8.0 |
| 36 | - | 3744933 | 3745106 | 174 | 50S ribosomal protein L32 | 7.9 |
| 37 | - | 5058593 | 5058838 | 246 | 50S ribosomal protein L31 type B | 7.8 |
| 38 | - | 5213716 | 5213949 | 234 | 30S ribosomal protein S18 | 7.8 |
| 39 | + | 125522 | 125722 | 201 | 50S ribosomal protein L29 | 7.7 |
| 40 | + | 4459692 | 4460294 | 603 | 30S ribosomal protein S4 | 7.7 |
| 41 | - | 4082555 | 4082704 | 150 | 50S ribosomal protein L33 | 7.6 |
| 42 | + | 127368 | 127553 | 186 | 30S ribosomal protein S14 | 7.6 |
| 43 | - | 4911264 | 4911806 | 543 | ribosomal subunit interface protein | 7.4 |
| 44 | - | 4380540 | 4380740 | 201 | 50S ribosomal protein L35 | 7.3 |
| 45 | + | 105572 | 106264 | 693 | 50S ribosomal protein L1 | 7.2 |
| 46 | + | 139149 | 139586 | 438 | 50S ribosomal protein L13 | 7.2 |
| 47 | - | 3656275 | 3656619 | 345 | 50S ribosomal protein L19 | 6.9 |
| 48 | - | 3658378 | 3658650 | 273 | 30S ribosomal protein S16 | 6.9 |
| 49 | + | 139608 | 140000 | 393 | 30S ribosomal protein S9 | 6.6 |
| 50 | - | 4380146 | 4380502 | 357 | 50S ribosomal protein L20 | 6.6 |
| 51 | + | 4136729 | 4136986 | 258 | 30S ribosomal protein S20 | 6.4 |
| 52 | - | 3639863 | 3640420 | 558 | ribosome recycling factor | 5.6 |

**Supplementary Table 4**. A list of 82 *Bacillus anthracis* transcriptional factor genes. The expression level under “Control” growth condition was determined from read coverage data. The list is sorted in descending order of the expression level.

| **Index** | **Strand** | **Left end** | **Right end** | **Length (nt)** | **Function** | **Expression level, log2(Control)** |
| --- | --- | --- | --- | --- | --- | --- |
| 1 | + | 161256 | 161987 | 732 | gluconate operon transcriptional repressor | 4.8 |
| 2 | - | 3940320 | 3940775 | 456 | FUR family transcriptional regulator | 4.6 |
| 3 | + | 4501738 | 4501959 | 222 | DeoR family transcriptional regulator | 4.6 |
| 4 | - | 1032181 | 1032738 | 558 | transcriptional regulator Hpr | 4.4 |
| 5 | - | 3669086 | 3669748 | 663 | fatty acid biosynthesis transcriptional regulator | 4.4 |
| 6 | - | 4411232 | 4411876 | 645 | GntR family transcriptional regulator | 4.4 |
| 7 | + | 1262307 | 1262963 | 657 | GntR family transcriptional regulator | 4.3 |
| 8 | - | 3144778 | 3145185 | 408 | transcriptional regulator, putative | 4.2 |
| 9 | - | 3930732 | 3931151 | 420 | MarR family transcriptional regulator | 4.1 |
| 10 | - | 2810798 | 2811187 | 390 | GntR family transcriptional regulator | 3.9 |
| 11 | - | 4130197 | 4131213 | 1017 | heat-inducible transcription repressor | 3.8 |
| 12 | + | 87415 | 87876 | 462 | transcriptional regulator CtsR | 3.8 |
| 13 | + | 104268 | 104801 | 534 | transcription antitermination protein NusG | 3.7 |
| 14 | + | 4509874 | 4510320 | 447 | CarD family transcriptional regulator | 3.5 |
| 15 | + | 2431275 | 2432723 | 1449 | GntR family transcriptional regulator | 3.5 |
| 16 | + | 1068049 | 1069026 | 978 | LacI family sugar-binding transcriptional regulator | 3.4 |
| 17 | + | 2997745 | 2998368 | 624 | ArsR family transcriptional regulator | 3.3 |
| 18 | - | 3604366 | 3605091 | 726 | GntR family transcriptional regulator | 3.3 |
| 19 | - | 4020592 | 4020984 | 393 | transcription antitermination protein NusB | 3.2 |
| 20 | - | 4329660 | 4330247 | 588 | TetR family transcriptional regulator | 3.2 |
| 21 | + | 2199004 | 2199303 | 300 | ArsR family transcriptional regulator | 3.1 |
| 22 | + | 607384 | 607761 | 378 | ArsR family transcriptional regulator | 3.0 |
| 23 | - | 1272208 | 1272660 | 453 | PadR family transcriptional regulator | 2.9 |
| 24 | - | 3866968 | 3867999 | 1032 | maltose operon transcriptional repressor | 2.8 |
| 25 | - | 4720714 | 4721445 | 732 | transcriptional activator tipA, putative | 2.8 |
| 26 | + | 256546 | 257175 | 630 | redox-sensing transcriptional repressor Rex | 2.8 |
| 27 | + | 612020 | 613288 | 1269 | transcriptional regulator | 2.8 |
| 28 | - | 3409929 | 3410405 | 477 | CarD family transcriptional regulator | 2.6 |
| 29 | + | 680011 | 680469 | 459 | MarR family transcriptional regulator | 2.4 |
| 30 | - | 4993371 | 4994282 | 912 | membrane-bound transcriptional regulator LytR | 2.4 |
| 31 | + | 1299031 | 1299693 | 663 | ExsB family transcriptional regulator | 2.3 |
| 32 | + | 3970305 | 3970751 | 447 | MarR family transcriptional regulator | 2.3 |
| 33 | + | 1882201 | 1882488 | 288 | transition state transcriptional regulatory protein | 2.1 |
| 34 | - | 1239560 | 1239883 | 324 | transcriptional regulator SinR | 2.0 |
| 35 | + | 54524 | 58054 | 3531 | transcription-repair coupling factor | 2.0 |
| 36 | - | 325522 | 326004 | 483 | MarR family transcriptional regulator | 1.9 |
| 37 | - | 798658 | 799854 | 1197 | LytR family transcription antiterminator | 1.9 |
| 38 | + | 626770 | 627057 | 288 | ArsR family transcriptional regulator | 1.9 |
| 39 | - | 2720254 | 2720820 | 567 | TetR family transcriptional regulator | 1.9 |
| 40 | - | 4387571 | 4388032 | 462 | transcriptional regulator NrdR | 1.8 |
| 41 | - | 4716217 | 4716849 | 633 | DeoR family transcriptional regulator | 1.7 |
| 42 | + | 4918477 | 4919604 | 1128 | LytR family transcription antiterminator | 1.7 |
| 43 | - | 2851347 | 2852099 | 753 | IclR family transcriptional regulator | 1.5 |
| 44 | + | 3078567 | 3078983 | 417 | MarR family transcriptional regulator | 1.5 |
| 45 | - | 4097571 | 4097984 | 414 | FUR family transcriptional regulator | 1.5 |
| 46 | + | 1428723 | 1429598 | 876 | MerR family transcriptional regulator | 1.5 |
| 47 | + | 2314930 | 2315412 | 483 | MarR family transcriptional regulator | 1.5 |
| 48 | + | 1332058 | 1332453 | 396 | MarR family transcriptional regulator | 1.3 |
| 49 | + | 1621040 | 1621624 | 585 | TetR family transcriptional regulator | 1.3 |
| 50 | + | 1294274 | 1294720 | 447 | MarR family transcriptional regulator | 1.3 |
| 51 | - | 3522017 | 3522769 | 753 | DeoR family transcriptional regulator | 1.2 |
| 52 | - | 960734 | 961300 | 567 | TetR family transcriptional regulator | 1.2 |
| 53 | - | 3180750 | 3181487 | 738 | GntR family transcriptional regulator | 1.1 |
| 54 | - | 4035975 | 4036403 | 429 | manganese transport transcriptional regulator | 1.1 |
| 55 | - | 4507742 | 4508065 | 324 | ArsR family transcriptional regulator | 1.1 |
| 56 | + | 1988543 | 1988929 | 387 | GntR family transcriptional regulator | 1.1 |
| 57 | + | 4041442 | 4042017 | 576 | TetR family transcriptional regulator | 1.0 |
| 58 | + | 1825288 | 1825731 | 444 | MarR family transcriptional regulator | 1.0 |
| 59 | + | 1857046 | 1858047 | 1002 | LytR family transcription antiterminator | 1.0 |
| 60 | - | 2964970 | 2965389 | 420 | MarR family transcriptional regulator | 1.0 |
| 61 | - | 4663311 | 4663808 | 498 | AsnC family transcriptional regulator | 0.9 |
| 62 | - | 3950770 | 3951633 | 864 | LacI family sugar-binding transcriptional regulator | 0.8 |
| 63 | + | 1062697 | 1063269 | 573 | TetR family transcriptional regulator | 0.7 |
| 64 | - | 3042523 | 3042822 | 300 | ArsR family transcriptional regulator | 0.7 |
| 65 | + | 1313205 | 1314218 | 1014 | AbrB family transcriptional regulator | 0.7 |
| 66 | - | 3840914 | 3841771 | 858 | LysR family transcriptional regulator | 0.6 |
| 67 | - | 3906443 | 3907291 | 849 | transcription antiterminator GlcT | 0.5 |
| 68 | + | 3020155 | 3021144 | 990 | LacI family sugar-binding transcriptional regulator | 0.5 |
| 69 | - | 4617806 | 4618531 | 726 | GntR family transcriptional regulator | 0.4 |
| 70 | - | 4790476 | 4790886 | 411 | MarR family transcriptional regulator | 0.4 |
| 71 | + | 408264 | 408845 | 582 | TetR family transcriptional regulator | 0.4 |
| 72 | + | 1309443 | 1309823 | 381 | GntR family transcriptional regulator | 0.3 |
| 73 | - | 3053221 | 3053661 | 441 | AsnC family transcriptional regulator | 0.3 |
| 74 | + | 3041269 | 3041844 | 576 | TetR family transcriptional regulator | 0.2 |
| 75 | - | 3198505 | 3198780 | 276 | ArsR family transcriptional regulator | 0.2 |
| 76 | + | 2884612 | 2884989 | 378 | transcriptional regulator AnsR | 0.2 |
| 77 | - | 5170751 | 5171623 | 873 | TetR family transcriptional regulator | 0.1 |
| 78 | + | 2354361 | 2354966 | 606 | TetR family transcriptional regulator | 0.1 |
| 79 | - | 2571006 | 2572856 | 1851 | acetoin operon transcriptional activator, putative | 0.1 |
| 80 | + | 1090916 | 1091818 | 903 | AraC family transcriptional regulator | 0.0 |
| 81 | - | 2764500 | 2765249 | 750 | IclR family transcriptional regulator | 0.0 |
| 82 | + | 4729449 | 4729778 | 330 | PadR family transcriptional regulator | 0.0 |
