# Supplementary material for: Bacillus anthracis genome organization in light of whole transcriptome sequencing
Source: BMC Bioinformatics. 2010 Apr 29;11(Suppl 3):S10. doi: 10.1186/1471-2105-11-S3-S10 (PMC2863060; doi:10.1186/1471-2105-11-S3-S10)
Supplement: Additional file 2 — Codon frequencies in the genome wide set of genes and its subsets along with frequencies of cognate tRNA genes for each codon. The codon frequencies were calculated from the three sets of coding sequences, namely the whole genome, the 100 most highly expressed genes as observed from the read coverage data and the 37 homologs to the proteins used by Sharp et. al (1987). The "Weighted 100 genes" column shows the frequencies of codons adjusted by weights, expression levels of the 100 genes as determined from read coverage data. The 95 tRNA genes shown in "tRNA genes" column were assigned to codons by tRNAscan-SE. Codon frequencies are normalized to 1000. Numbers in bold font indicate the maximum frequencies/counts in a synonymous group. [file 1471-2105-11-S3-S10-S2.doc]

Additional file 2.

| **Amino acid** | **Codon** | **Genomic Freq** | **100 genes** | **Weighted 100 genes** | **37 genes (CAI)** | **tRNA genes** | **Amino acid** | **Codon** | **Genomic Freq** | **100 genes** | **Weighted 100 genes** | **37 genes (CAI)** | **tRNA genes** | **Amino acid** | **Codon** | **Genomic Freq** | **100 genes** | **Weighted 100 genes** | **37 genes (CAI)** | **tRNA genes** | **Amino acid** | **Codon** | **Genomic Freq** | **100 genes** | **Weighted 100 genes** | **37 genes (CAI)** | **tRNA genes** |
| --- | --- | --- | --- | --- | --- | --- | --- | --- | --- | --- | --- | --- | --- | --- | --- | --- | --- | --- | --- | --- | --- | --- | --- | --- | --- | --- | --- |
| **Phe** | **TTT** | **32.8** | 10.2 | 9.3 | 5.1 | 0 | **Ser** | **TCT** | **15.5** | **23.5** | **24.1** | **26.1** | 0 | **Tyr** | **TAT** | **28.0** | 10.1 | 9.2 | 6.2 | 0 | **Cys** | **TGT** | **6.3** | **3.4** | **2.8** | **2.9** | 0 |
|  | **TTC** | 14.4 | **23.6** | **22.7** | **21.6** | **4** |  | **TCC** | 3.2 | 1.1 | 1.1 | 0.3 | 1 |  | **TAC** | 9.3 | **15.5** | **14.8** | **14.6** | **2** |  | **TGC** | 2.1 | 1.4 | 1.3 | 2.0 | **1** |
| **Leu** | **TTA** | **49.9** | **42.1** | **40.1** | **38.2** | **2** |  | **TCA** | 14.7 | 8.9 | 8.6 | 7.3 | **4** | **Stop** | **TAA** | 0.0 | 0.0 | 0.0 | 0.8 | 0 | **Stop** | **TGA** | 0.0 | 0.0 | 0.0 | 0.3 | 0 |
|  | **TTG** | 9.2 | 3.2 | 2.9 | 1.1 | 1 |  | **TCG** | 4.6 | 1.3 | 1.2 | 0.9 | 0 | **Stop** | **TAG** | 0.0 | 0.0 | 0.0 | 0.6 | 0 | **Trp** | **TGG** | **10.4** | **6.2** | **5.2** | **4.0** | **2** |
| **Leu** | **CTT** | 18.2 | 22.7 | 22.8 | 26.2 | 0 | **Pro** | **CCT** | 9.1 | 9.9 | 10.6 | 11.5 | 0 | **His** | **CAT** | **16.4** | **8.6** | 7.7 | 7.8 | 0 | **Arg** | **CGT** | **14.1** | **36.8** | **40.7** | **51.2** | **3** |
|  | **CTC** | 4.2 | 0.8 | 0.8 | 0.2 | 1 |  | **CCC** | 1.1 | 0.2 | 0.3 | 0.2 | 0 |  | **CAC** | 4.8 | 8.1 | **8.3** | **9.6** | **2** |  | **CGC** | 4.8 | 9.5 | 10.0 | 12.4 | 0 |
|  | **CTA** | 10.6 | 8.5 | 7.6 | 6.4 | 2 |  | **CCA** | **16.5** | **23.2** | **22.1** | **20.2** | **3** | **Gln** | **CAA** | **30.3** | **30.7** | **30.0** | **31.3** | **4** |  | **CGA** | 5.4 | 1.2 | 0.9 | 1.6 | 0 |
|  | **CTG** | 3.3 | 1.9 | 1.7 | 1.2 | 0 |  | **CCG** | 7.5 | 2.9 | 2.5 | 1.6 | 0 |  | **CAG** | 6.7 | 3.7 | 4.0 | 2.5 | 0 |  | **CGG** | 1.3 | 0.1 | 0.0 | 0.3 | 1 |
| **Ile** | **ATT** | **50.8** | 30.5 | 27.6 | 24.5 | 0 | **Thr** | **ACT** | 12.4 | 26.0 | **29.9** | **28.4** | 0 | **Asn** | **AAT** | **32.9** | 14.8 | 14.2 | 11.0 | 0 | **Ser** | **AGT** | 14.5 | 5.9 | 4.8 | 4.5 | 0 |
|  | **ATC** | 13.4 | **32.7** | **35.3** | **36.3** | **4** |  | **ACC** | 2.5 | 0.7 | 0.6 | 0.3 | 1 |  | **AAC** | 13.2 | **28.6** | **28.7** | **27.2** | **5** |  | **AGC** | 5.8 | 6.5 | 6.2 | 4.8 | 2 |
|  | **ATA** | 16.9 | 1.8 | 1.3 | 1.4 | 0 |  | **ACA** | **27.9** | **28.4** | 26.9 | 25.1 | **4** | **Lys** | **AAA** | **56.1** | **62.8** | **66.7** | **72.0** | **5** | **Arg** | **AGA** | 9.4 | 3.1 | 3.3 | 4.2 | 1 |
| **Met** | **ATG** | **25.0** | **24.7** | **22.9** | **21.4** | **8** |  | **ACG** | 13.5 | 5.4 | 5.3 | 3.9 | 0 |  | **AAG** | 18.1 | 14.1 | 15.4 | 17.7 | 0 |  | **AGG** | 2.4 | 0.2 | 0.2 | 0.6 | 0 |
| **Val** | **GTT** | 25.9 | 38.5 | 41.1 | **42.8** | 0 | **Ala** | **GCT** | 21.0 | **41.9** | **46.2** | **54.6** | 0 | **Asp** | **GAT** | **37.8** | **29.8** | **28.3** | **27.2** | 0 | **Gly** | **GGT** | **24.9** | **49.1** | **51.3** | **47.5** | 0 |
|  | **GTC** | 5.7 | 2.7 | 2.4 | 1.6 | 1 |  | **GCC** | 4.0 | 1.4 | 1.3 | 0.8 | 0 |  | **GAC** | 8.8 | 18.9 | 19.7 | 21.9 | **6** |  | **GGC** | 8.4 | 12.2 | 11.6 | 11.3 | **4** |
|  | **GTA** | **31.1** | **43.3** | **43.6** | 39.6 | **5** |  | **GCA** | **29.7** | 38.5 | 35.1 | 32.6 | **5** | **Glu** | **GAA** | **57.2** | **63.2** | **63.8** | **66.3** | **7** |  | **GGA** | 24.4 | 18.6 | 17.8 | 16.1 | **4** |
|  | **GTG** | 10.5 | 5.8 | 5.9 | 6.5 | 0 |  | **GCG** | 13.1 | 9.9 | 8.8 | 8.7 | 0 |  | **GAG** | 18.5 | 17.9 | 18.4 | 21.6 | 0 |  | **GGG** | 9.5 | 2.7 | 2.2 | 1.7 | 0 |
