# Supplementary material for: Bacillus anthracis genome organization in light of whole transcriptome sequencing
Source: BMC Bioinformatics. 2010 Apr 29;11(Suppl 3):S10. doi: 10.1186/1471-2105-11-S3-S10 (PMC2863060; doi:10.1186/1471-2105-11-S3-S10)
Supplement: Additional file 3 — Supplementary tables. [file 1471-2105-11-S3-S10-S3.doc]

**Supplementary Figures**

| **a)** | **b)** |
| --- | --- |
|  | |
